# Breaking down the fences among registries on autoinflammatory diseases: the E-Merge project

**DOI:** 10.1186/s13023-023-02812-4

**Published:** 2023-07-17

**Authors:** Y. Vyzhga, V. Hentgen, R. Caorsi, H. Wittkowski, M. Hofer, N. Ruperto, E. Lainka, K. Theodoropoulou, D. Foell, E. Mosci, M. Gattorno, AID-Net, Eurofever, JIRcohort registriesthe Paediatric Rheumatology International Trials Organisation (PRINTO)

**Affiliations:** 1 National Pirogov Memorial Medical University, Vinnytsya, Ukraine; 2 Department for Pediatrics, National Referral Centre of Auto-Inflammatory Diseases and Inflammatory Amyloidosis - CEREMAIA, Versailles Hospital, Le Chesnay, Paris, France; 3 Centre for Autoinflammatory Diseases and Immunodeficiencies, IRCCS Istituto Giannina Gaslini, Genoa, Italy; 4 Department of Pediatric Rheumatology and Immunology, University Hospital Munster, Münster, Germany; 5 Department of Pediatrics, Centre Hospitalier Universitaire Vaudois (CHUV), Lausanne, Switzerland; 6 University Hospital of Geneva, Geneva, Switzerland; 7 Gaslini Trial Centre, IRCCS Istituto Giannina Gaslini, Genoa, Italy; 8 Department of Pediatric Rheumatology, University Children’s Hospital Essen, Essen, Germany

**Keywords:** Autoinflammatory diseases, Disease registry, Eurofever, JIR-cohort, AID-Net

## Abstract

**Background:**

Among the various numbers of different autoinflammatory diseases (AIDs), the absolute majority of them remains rare, with a single representative in large populations. This project, endorsed by PRES, supported by the EMERGE fellowship program, and performed in line with the Metadata registry for the ERN RITA (MeRITA), has the objective of performing a data synchronization attempt of the most relevant research questions regarding clinical features, diagnostic strategies, and optimal management of autoinflammatory diseases.

**Results:**

An analysis of three large European registries: Eurofever, JIR-cohort and AID-Net, with a total coverage of 7825 patients from 278 participating centers from different countries, was performed in the context of epidemiological and clinical data merging. The data collected and evaluated in the registries does not cover only pediatric patients, but also adults with newly diagnosed AIDs. General aspects of the existing epidemiological data have been discussed in the context of patient global distribution, potential diagnostic delays, access to genetic testing, and the availability of the treatment.

**Conclusions:**

In general, the results indicate a great potential for upcoming collaborative work using existing data in cohorts that enhance the quality of medical care performed for patients with autoinflammatory diseases.

**Supplementary Information:**

The online version contains supplementary material available at 10.1186/s13023-023-02812-4.

## Background

Systemic autoinflammatory diseases (SAIDs) are a cluster of monogenic and polygenic inborn/acquired inflammatory diseases associated with dysregulations in the innate immune system [1]. The first international series of patients that were affected by genetically determined conditions such as Familial Mediterranean Fever (FMF), TNF receptor 1-associated periodic syndrome (TRAPS), and mevalonate kinase deficiency (MKD) have been described from 1999 onwards [2, 3].

The first formal international registry for an autoinflammatory disease was established in the Netherlands to collect clinical and laboratory data on Hyper-IgD syndrome, which then transformed into HIDSnet. Information from this registry allowed us to summarize the existing clinical knowledge, define treatment algorithms, and provide data on the follow-up of the patients. Similarly, an international European consortium (EUROTRAPS) was developed to collect data from six different countries (Austria, France, Germany, Israel, Italy, and the United Kingdom) to gain insights about TRAPS [4, 5]. In 2008, M. Hofer established a web-based multicentric registry for PFAPA as an international collaboration in the context of the working party “periodic fevers” of Pediatric Rheumatology European Society (PRES) [6]. Data from the above registries provided pivotal information on these newly discovered conditions. In 2009, Ben-Chetrit and Touitou launched an international cohort of FMF patients aimed at defining the epidemiological, genetic, and clinical characteristics of FMF [7].

The great increase in the number of SAIDs in the last 20 years, highlighted the limits of carrying unique registries for each condition, thus prompting the need for establishing common registries that can enroll patients with different diseases and are flexible enough to include the newly identified conditions [3, 8, 9].

The Eurofever registry, a multi-national clinical registry hosted by the Pediatric Rheumatology International Trials Organization (PRINTO), has been held in the context of the PRES since 2009 [10, 11]. Around the same time, a German national database for autoinflammatory diseases, the AID-NET, was established [12]. This registry gathered information and samples from German patients with AIDs for 10 consecutive years up until 2018. In the year 2013, the JIR Cohort, a multicenter prospective data repository for patients with systemic inflammatory or rheumatological disease, was created in the context of francophone countries and afterwards extended to other countries. Also, a specific module dedicated to AIDs was developed in 2016 where patients from the main centers of expertise for autoinflammatory diseases in France and Switzerland have been enrolled [13].

It is worth mentioning that although every register has its own structure, yet merging the collected data would be useful to obtain a more robust body of evidence on these rare conditions. And in order to synchronize the data and perform a comparative analysis of the most relevant research questions, a research fellowship was endorsed by the PRES working party on autoinflammatory diseases to improve further collaborative work in the registries.

The current E-Merge project aimed to test the possibility of group analysis of data collected from different registries, analyzing the strengths and limitations of this approach.

## Materials and methods

In order to achieve the aim of the current project, the three registries (Eurofever, JIRcohort, and AID-net), which collect information on AID patients in Europe and extra-European countries discussed a list of variables subject to the evaluation of the current study, in a consensus meeting that was held in Chateau d’Oex (Switzerland) in October 2021 (Additional file 1: Table S1).

The basic set of epidemiological data was extracted anonymously by each coordinator, provided, and analyzed as aggregated data (e.g., no individual patient's data was provided).

Each registry representative provided information about cohort design, duration and procedure of patient follow-up, chapters about main epidemiological, clinical, and laboratory information, data collection methods, the process of IT support, etc. This study also includes descriptive reviews of published articles and studies based on registry data.

Moreover, a formal primary hypothesis or statistical testing has not been provided, since this study has a descriptive character. Categorical data were reported in terms of absolute frequencies and percentages. Continuous data has been described in terms of Mean, Median, Minimum and Maximum, and 1st and 3rd quartiles (IQR). IBM SPSS (SPSS Inc. Chicago, IL, 29.0) and excel have been used for descriptive statistics.

The data analysis has been conducted by YV during a 6-month PReS EMERGE fellowship, with attendance at CHUV Lausanne (Switzerland), IRCCS Istituto Giannina Gaslini in Genoa, and UKM in Muenster (Germany). Each attendance duration lasted for 2 months, respectively.

### Origin and structure of the three registries

#### Eurofever

The Eurofever project was promoted in 2008 by the SAIDs working group of the PRES and supported by the Executive Agency for Health and Consumers (EAHC, project 2007332). The electronic data capture (EDC) system of the registry was developed by PRINTO in November 2009. All centres belonging to the PRINTO network dealing with AIDs were offered the possibility of participating in the registry. In addition, adult centres managing SAIDs were also invited to participate.

The original structure of the registry consisted of two parts: demographic information and clinical manifestations. In 2015, a protocol amendment updated the registry with the new AIDs and the elaboration of a longitudinal observational part.

The quality control applied to the registry consists of a routine check of the completeness and coherence of data by the PRINTO staff; if any relevant query is raised during the check, a question is addressed to the investigator to confirm or modify the data. Also, the collection of the information has a longitudinal prospective design with regular onsite supervision by PRINTO technical support. The registry continues its recruitment activity with a collection of demography and clinical information on AIDs.

#### AID-Net

In 2009, the research initiative AID-Net (Network for Inflammatory Diseases), funded by the German Federal Ministry of Education and Research (BMBF project 01GM08104), was established. The network was made up of five basic research projects and three clinical research projects located at 12 institutions all over Germany. The main unit of the collaborative clinical research core of AID-Net was the patient registry.

Recruitment of patients with autoinflammatory diseases was prompted via AID-Net (an online registry), and patient material was collected and stored in a central biomaterial bank for DNA and serum. Data from the registry and the biobanks is connected to the online user interface which allows the patient’s biomaterial to be used to identify genetic or serological markers of AIDs.

All members of the German Society for Pediatric Rheumatology (GKJR) were invited to participate in the project. Furthermore, immunological and molecular genetics laboratories had access to the online system for sample management documentation. The online registry was operated by EDC with ease. All data were entered via remote data entry software ProMISe (Project Manager Internet Server) version 2.0, which had been developed by R Brand. Technical and operating support for the registry was provided by Sabrina Fühner and Elke Lainka. Data checks and validation were provided on demand within particular scientific projects.

#### JIR cohort

The JIR cohort is an initiative of Belgian, French, and Swiss pediatric rheumatologists that was endorsed by the scientific societies “SOFREMIP” (French-speaking pediatric rheumatology society) and “PRS” (Pediatric Rheumatology Switzerland). The registry was launched in 2013 and designed for patients suffering from rheumatic diseases with childhood onset. Its primary goal was to improve patients' care through organizing and evaluating the outcomes of pediatric rheumatic diseases. The initial financial support for the creation of the JIR platform was the “Aquira Award” in 2013. The first 24 participating centres were recruited through the SOFREMIP and PRS networks between 2013 and 2016. Moreover, since 2016, a bilingual French–English interface has been implemented, allowing non-French-speaking centres to participate in the registry.

Initially, the registry was composed of three managing committees: steering, scientific, and executive, with members representing the participating countries. The registry is currently hosted by the Foundation RES, whose goals are to support research and training in pediatric rheumatology.

The registry data are collected prospectively by the investigating physician or clinical research technician, then implied in the register, where the register itself serves as a medical record in a few cases. The registry is composed of a common core for all diseases and specific modules collecting disease-specific data (autoinflammatory diseases, systemic onset JIA, JIA, uveitis, lupus). All the data before 2013 was collected retrospectively; since then, the register has been operating as a longitudinal observational cohort.

The registry works on “OCQMS,” a software developed by Swiss IT Company Seantis Gmbh, which is already successfully used by the Swiss adult rheumatology registry (SCQM). And technical support is provided by Seantis GmbH. Data cross-checking is performed when the data is exported for scientific purposes and is usually performed either by the clinical researcher performing the project or by the physician requesting the data, depending on the particular situation.

All participating centres in the three mentioned registries received approval from the related ethics committee, and consent or assent has been obtained by patients and their families.

## Results

**Fig. 1:**
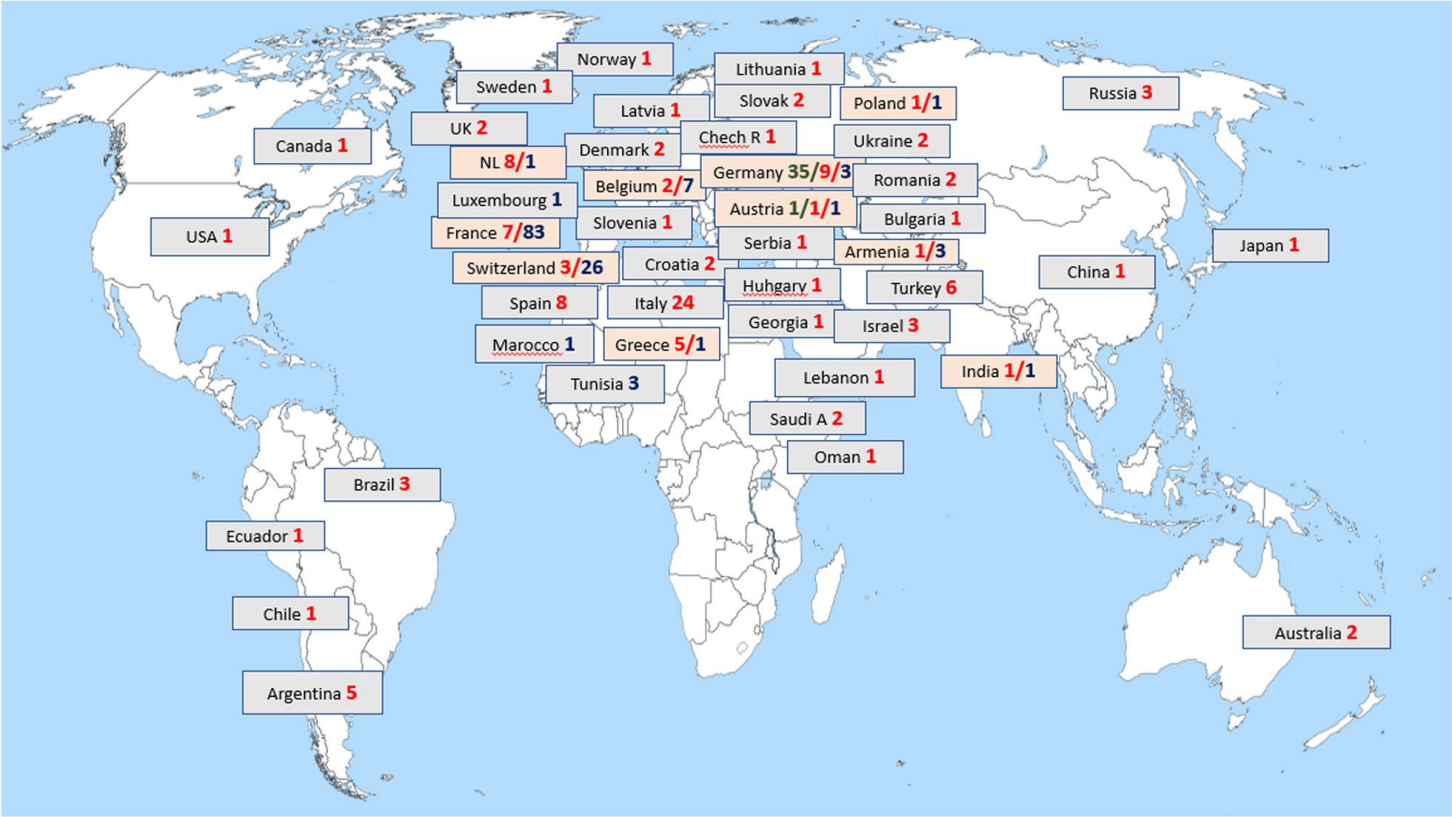
Overview of centres participating in Eurofever, JIRcohort and AID-Net. *Marked red—centres participating in Eurofever. Marked blue—centres participating in JIRcohort. Marked green —centres participating in AID-Net. Textboxes colored orange—potential overlap between the patients in different registries

In total, the 3 registries cover 7825 patients with different AIDs from 278 participating centres from different parts of the world (Fig. 1).

AID-Net is a national registry involving 36 main pediatric rheumatology centers in Germany. JIR and Eurofever are multi-national cohorts, covering about 40 countries in total. Data collection in AID-Net was completed in 2018, but JIR and Eurofever continue active recruitment and follow-up on patients previously included. The information summarized in the registries covers not only pediatric patients but also adults with newly diagnosed AIDs and a different proportion of the previously diagnosed ones (Table 1).

**Table 1:** Demography and epidemiological data of the patients with autoinflammatory diseases presented in registries

	JIR cohort (n = 1897)	Eurofever (n = 4552)	AID-Net (n = 1375)
Number of the participating countries	13	42	2
Number of the centres	125	117	36
Number of autoinflammatory diseases recorded	18	32	10
Male/female number	947/950	2237/2315	690/684
Adults > 18 y.o. diagnosed with AID	412 (21.7%)	784 (17.2%)	28 (2.0%)
Children < 18 y.o. diagnosed with AID	1485 (78.3%)	3768 (82.8%)	1324 (96.3%)
Unknown			23 (1.7%)
*Number of patients enrolled within time-period*			
2009–2012	2 (0.1%)	1517 (33.3%)	534 (38.8%)
2013–2016	569 (30.0%)	774 (17.0%)	632 (46.0%)
2017–2021*	1326 (69.9%)	2261 (49.7%)	209* (15.2%)
*Number of patients receiving their diagnosis within time-period*			
Before 2009	412 (21.7%)	1641 (36%)	366 (26.6%)
2009–2012	240 (12.7%)	1460 (32.1%)	383 (27.9%)
2013–2016	490 (25.8%)	855 (18.8%)	495 (36.0%)
2017–2021*	755 (39.8%)	596 (13.1%)	131 (9.5%)
Age of AID diagnose	7.21 (0.56–71.8)	11.0 (0.18–66.2)	6.3 (0.17–56.1)
Children (< 18 y.o.)	5.5 (0.08–17.99)	5.4 (0.1–17.4)	6.3 (0.18–18)
Adults (> 18 y.o.)	31.66 (18.02–71.8)	39.1 (20.0–66.2)	29.0 (18.1–56.1)
*Number of the patients enrolled within different AID*			
FMF	775 (40.8%)	1363 (29.9%)	587 (42.6%)
PFAPA	456 (24%)	676 (14.8%)	140 (10.2%)
SURF	312 (16.4%)	429 (9.4%)	83 (6.0%)
MKD	55 (2.9%)	221 (4.8%)	8 (0.5%)
CAPS	103 (5.4%)	288 (6.3%)	41 (2.9%)
TRAPS	56 (2.9%)	287 (6.3%)	47 (3.4%)
CRMO	94 (4.9%)	597 (13.1%)	193 (14.0%)
SJIA	392 (20.7%)	6 (0.1%)	262 (19%)
Behcet disease	97 (5.1%)	282 (6.2%)	N/A

*Data cut-off as on September 2021

The number of conditions collected by each registry was rather variable. Common information was available for 10 diseases only: FMF, PFAPA, SURF (Undefined Recurrent Fever), CRMO, SoJIA, CAPS, TRAPS, and MKD. Eurofever and JIR cohort display a much larger overlap of rare conditions collected by both registries (Additional file 2: Table S2).

The distribution of patients among registries was not even. Of the 125 centers participating in the JIR cohort, 23 centers from Switzerland and France enrolled more than 90% of all patients. From the Eurofever register, patients were scattered and not focused in one part of Europe, with 43% of patients from centers in Southern Europe, 26% from Western Europe, 8.7% from Northern Europe, and 9% from the Middle East, with a tendency toward lower incidence in Eastern Europe countries (Additional file 1: Fig. S1).

The evaluation of patient ethnicity revealed a predominance of Caucasians in all registries, with up to 20% of others not immediately definable. However, the way ethnicity data was collected among the three registries was rather heterogeneous, representing a possible major limitation for a homogenous analysis (Table 2).

**Table 2:** Ethnicity of the patients included in AID-Net, Eurofever, JIR cohort

Ethnicity	JIRcohort (n = 1897)	Eurofever (n = 4552)	AID-Net (n = 1376)
Caucasian (white)	1352 (71.3%) Caucasian-603 Mediterranean basin-749	4064 (89.3%) Caucasian European-3429 Caucasian Indian-5 Caucasian Middle East-569 Caucasian North African-61	1094 (79.5%) German Caucasian-577 Turkish-415 Arab-46 Armenian-10 Italian-9 Kazakh-7 Greek-5 Caucasus-5 Albanian-4 Kurd-4 Romanian-4 Slavic-3 Egyptian-2 Baltic-1 Portuguese-1 Azerbaijan-1 Asyric-2 Persian-2
American Indian	1 (0.05%) North central American-1	1 (0.02%) Native American-1	0
Black/African American	15 (0.7%) African west Indian-6 Subsaharian African-9	46 (1%) African-46	1 (0.07%) Berber Sahara-1
Asian	22 (1.6%) Asia-22	62 (1.36%) North East Asian-40 South East Asian-22	1 (0.07%) Vietnamese-1
Latino	0	27 (0.5%) Hispanic-27	0
Native Hawaiian	1 (0.05%) Oceania-1	0	0
	Mixed-143 (7.5%) Unknown-20 (1%) N/A-343* (18%)	Mixed-238 (5.2%) Other-31 (0.6%) Cannot report-83 (1.8%)	Unknown-274 (20%)

Not a mandatory question to be completed in the registry; ethnicity listed as it mentioned in the registry and defined by physician

**Fig. 2:**
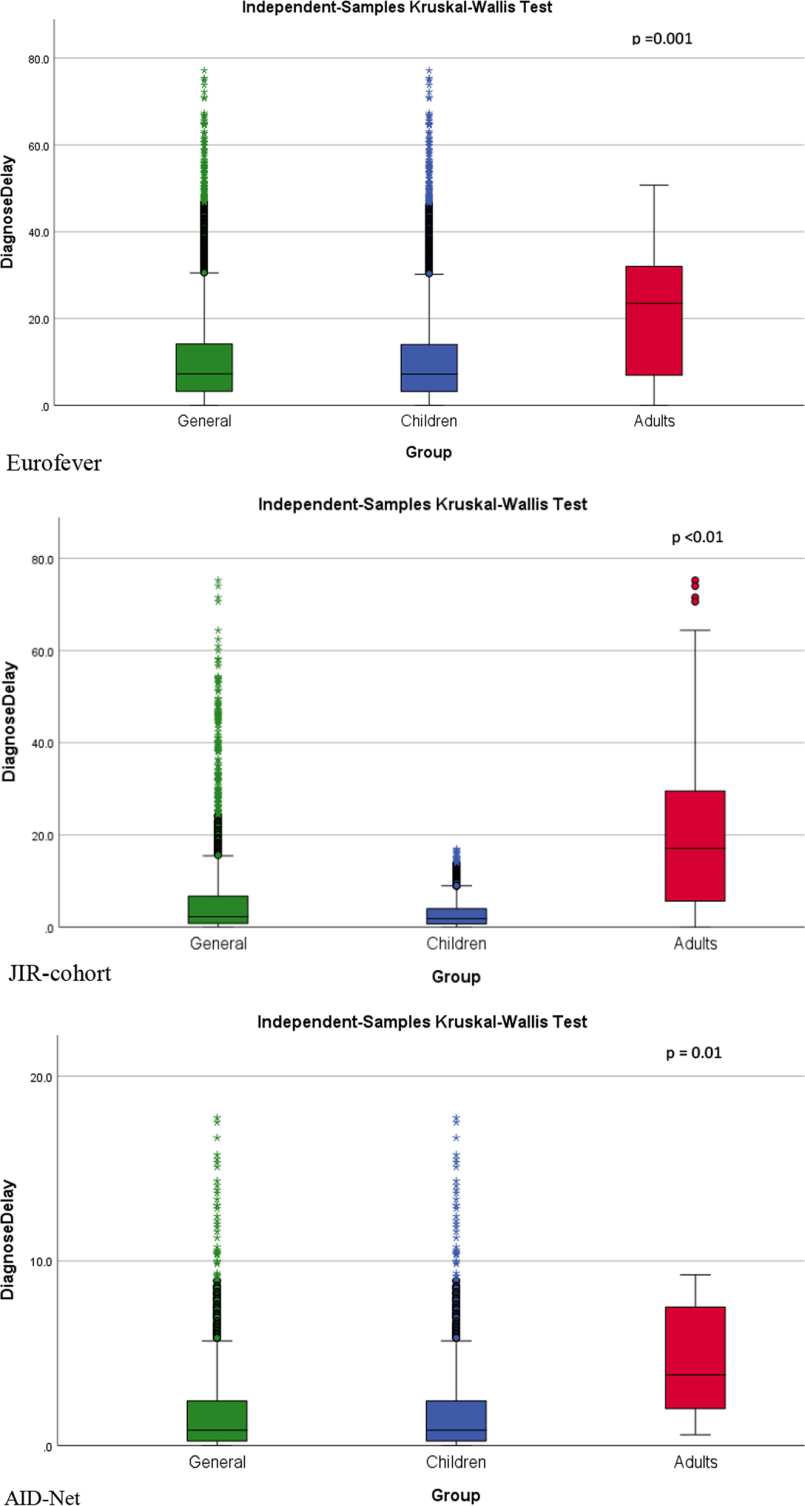
Diagnostic delay (years) in patients with autoinflammatory diseases in general cohort, children and adults throughout the registries

In contrast, the mode of collecting demographic data in the three registries was certainly found to be rather homogeneous, allowing, for example, a comparison of the diagnostic delay found in the three registries for different diseases. In all registries, a significant delay between disease onset and diagnosis was noticed, with a much shorter trend in children (Fig. 2). The highest rate of diagnostic delay was observed for the "classical" monogenic AIDs, such as TRAPS, MKD, and CAPS, that displayed the higher percentage of adult patents. Notably in many of them, the disease onset occurred after the causative gene had already been identified (Table 3).

**Table 3:** Diagnostic delay (Median (ql—q3), years) in patients with the most common AIDs presented in AID-Net, Eurofever, JIR cohort

	JIR (n = 1897)	Eurofever (n = 4552)	AID-Net (n = 1376)
FMF	2.99 (0.1–45.2)	2.52 (0.0–13.96)	1.66 (1.29–2.05)
PFAPA	1.99 (0.0–40.97)	1.52 (0.0–11.79)	1.63 (1.25–2.01)
SURF (Undefined recurrent fever)	2.21 (0.4–48.4)	2.44 (0.1–52.3)	0.58 (0.31–1.29)
CAPS	10.31 (0.0–74.1)	7.12 (0.1–67.4)	2.17 (0.83–3.51)
MKD	4.75 (0.4–54.39)	5.48 (0.3–62.6)	0.91 (0.65–3.06)
TRAPS	11.26 (0.7–60.95)	13.31 (1.2–46.5)	1.01 (0.35–1.68)
CNO	0.54 (0.1–9.91)	1.49 (1.3–15.3)	0.58 (0.35–0.82)
soJIA	0.11 (0.02–2.11)	0.17 (0.0–1.2)	0.08 (0.02–0.29)

The information concerning the genetic testing was also analyzed in details (Table 4). More than 8000 genetic tests have been reported in registries, with a detailed information concerned the methods used for the analysis (Sanger, NGS panel, whole exome sequencing, whole genome sequencing).

**Table 4:** Types of genetic tests reported in patients with different autoinflammatory diseases recorded in JIR-cohort, Eurofever and AID-Net

	JIR (n = 1897)	Eurofever (n = 4552)	AID-Net (n = 1376)
Number of patients with reported genetic testing	1172 (61.7%)	2605 (57.2%)	735 (53.4%)
Number of the tests performed	1687	5955	735
*Method used*			
Sanger	1003 (59.4%)	2763 (41.1%)	415 (56.4%)
Targeted exome sequencing	7 (0.4%)	N/A	88 (11.9%)
Whole exome sequencing (WES)	21 (1.2%)	174 (2.4%)	23 (3.1%)
Whole genome sequencing (WGS)	193 (11.4%)	1463 (21.8%)	N/A
Testing method unknown	463 (27.4%)	463 (6.9%)	209 (28.4%)

We report the main similarities and differences found in the three registry for the collection of the clinical data. All three registries presented a homogeneous approach for the collection of the most relevant clinical features (characteristic of fever episodes, clinical manifestations according to different organs and systems, laboratory examination and treatment) associated to AIDs. Some distinctive information has been also identified for each registry (Table 5).

**Table 5:** Differences and similarities among the items collected in three registries

	Similarities	Specific for JIRcohort	Specific for Eurofever	Specific for AID-Net
Characteristic of the disease episode	Pattern of the regularity Duration of the episode Number of the episodes/year Triggers	Frequency of the attacks before therapy Prodromes Seasonality	Seasonality	
Organs and systems clinical manifestation	Muco-cutaneous manifestations Musculoskeletal system Gastrointestinal system Lymphoid organs Cardio-vascular respiratory system Neurological symptoms Other organs and systems	Presence of the symptom during and between attack in a quality type of an answer (yes/no)	4 variants of quality answer (never/sometimes or often/always/unknown)	Quality type of an answer (yes/no)
Laboratory examinations and genetic testing	Routine blood examinations Urine examinations Specific tests Results of additional investigations (optional) Genetic testing results	Test results Format of genetic testing is not unified	Quality answers (normal/abnormal /not done) Unified genetic testing format	Test results Unified genetic testing format
Drug therapy	Start date End date Dose/frequency/route Safety	Drugs to treat AID and comorbid conditions	Treatment compliance	
Quality of life		DAS physician/patient	DAS physician/patient AIDAI Quality of life	
Others	Family background	App for patients	Optional part with other modules Tanner stage, infection assessment, prothrombotic markers, lung functional test, stem cell transplantation, imaging and other diagnostical procedures	Possibility to enter clinical data in 3 months and 1 week before the visit

Finally, all three registries allowed the collection of detailed information on the main drugs used in the field of AID (colchicine, DMARDS, biologics, steroids), with the possibility to specify the duration of the treatment, the dose and the frequency of administration and possible adverse events. An example of the possible evaluation of the treatment strategies used in the three registry is provided, showing a rather homogeneous distribution of the information (Table 6).

**Table 6:** Types of drugs used and number of patients reported in registries

	JIR cohort (n = 1897)	Eurofever (n = 2417)*	AID-Net (n = 1376)
Colchicine	838 (44.2%)	848 (35.1%)	597 (43.4%)
Steroids	561 (29.6%)	737 (30.5%)	497 (36.1%)
*Biologics*			
Anakinra	151 (7.9%)	242 (10.0%)	188 (13.7%)
Canakinumab	84 (4.4%)	162 (6.7%)	83 (6.0%)
Adalimumab	43 (2.3%)	82 (3.4%)	54 (3.9%)
Etanercept	26 (1.4%)	79 (3.2%)	131 (9.5%)
Infliximab	24 (1.3%)	32 (1.3%)	17 (1.2%)
Tocilizumab	5 (0.2%)	13 (0.5%)	46 (3.3%)

*Complete data concerning treatment options available for patients inserted after 2015

## Discussion

In this study, we provide evidence on the actual possibility of merging demographic, genetic, and clinical data from different registries on rare conditions, taking as an example three “historical” registries in the context of autoinflammatory diseases. The study explores the degree of homogeneity among the three registries and the possibility of retrieving scientifically relevant information from their demographic parts.

Many critically important questions arise concerning the clinical features, diagnostic strategy, and optimal management of AIDs. Due to the low incidence of the diseases, no single registry could answer all these questions. Also, all registries must follow a defined purpose that reflects the process of data and item collection [14–17].

We were able to show in this study that although these registries for auto-inflammatory diseases were developed independently in specific contexts and countries, they still present important homogeneity in the variables recorded. All of them include the main epidemiological data (with a focus on the age, gender, date of diagnose and diagnostic delay), a similar clinical part involving manifestations from the main systems and organs, part of the laboratory investigations, genetic testing availability, and treatment. The difficulties of data merging is demonstrated in the example of ethnicity characteristics (Table 2) and proves that the method of data collection may significantly influence the final outcome. At the same time, it describes potential differences in the sort of data collection among the registries.

As the result of the reclassification of some data, it has been evident that merging information from different registries is possible and provides cohorts with many patients, even for ultra-rare diseases (Additional file 2: Table S2). The main challenge associated with AIDs is their rarity, which makes it difficult to enroll patients and collect data, particularly when evaluating treatment strategies for new medications or performing cohort studies that require merging data from multiple sources. Despite these challenges, two out of the three registries included in the study continue to actively recruit patients. Additionally, the biobank of AID-Net is also available, which can be considered as an active registry. The study found that the majority of the centers with high enrollment rates are located in Western and Southern Europe, while the Eastern part of Europe is less involved. This highlights the potential for further cooperation and addressing the issue of underdiagnosis of AIDs in those regions, as well as the possibility of low activity in specialized centers. Additionally, there is currently limited involvement from important geographic areas in North America and Asia, but their participation could potentially increase the number of recruited patients and enable more global studies on AIDs.

Challenges:

The following challenges are encountered in conducting similar projects:

- It is important to address the issue of potential multiple entries of the same patient into different registries when merging data from different sources. While the distribution of centers in different regions may potentially help identify such cases, it is not sufficient on its own. Mechanisms like the MeRITA system used by the ERN RITA project can be employed to automatically detect and exclude duplicated [18]. It is important to discuss such issues with IT support and at the site level before collaborative studies to ensure accurate data analysis. Additionally, it should be noted that encrypted information may hinder manual checks and other measures may need to be employed to detect duplicates.
- Analyzing the data in a detailed manner can be challenging due to differences in granularity across different registries. As a result, it may be difficult to answer complex questions regarding specific clinical signs or describe the presence of specific tests. However, combining data from multiple registries can increase the statistical power of the results. It is important to be cautious when merging data, as similar viewpoints may not necessarily lead to similar outcomes. Differences in the methods of outcome ascertainment, such as the collection of clinical information, symptom details, results of investigations, and treatment, can significantly impact results even if the primary working model is similar. Standardization or unification of clinical data across AID registries can help overcome these problems and facilitate data merging in the future.
- Longitudinal data collection will allow for the identification of potential risk factors, prognostic factors, and the natural history of the disease. Furthermore, longitudinal studies can also help identify changes in disease patterns over time, such as the emergence of new clinical phenotypes or changes in treatment response. It is important to standardize the follow-up protocol and data collection methods to ensure that the collected data can be accurately compared and combined between different studies and cohorts. This will require collaboration and cooperation between different centers and networks to establish a standardized follow-up protocol for AIDs.
- Aggregating data from different registries into tables can help overcome limitations related to obtaining informed consent from individual patients, but it may also limit the ability to perform more complex statistical analyses. It is important to consider differences in healthcare systems and clinical expertise when interpreting the results of such studies. Collaborative studies can help identify these differences and potential pitfalls on a geographical and economic level, leading to improvements in the quality of medical care in the future.

## Conclusions

It is clear that collaborative efforts are necessary to overcome the challenges of studying rare diseases like AIDs. Despite limitations in data collection and analysis, this descriptive overview of three historical AIDs registries in Europe highlights the potential for further collaboration and optimization of data collection. Standardization of clinical data and longitudinal follow-up monitoring will be important for future studies, and mechanisms like the MERITA system [18] can help address the issue of potential duplicate data. By working together, researchers and healthcare providers can improve the quality of care for patients with AIDs and advance our understanding of these rare diseases.

## Supplementary Information

**Additional file 1: Table S1** Variables, suggested to be analysed from the registries (according to final meeting decision). **Figure S1** Distribution of patients with autoinflammatory diseases within regions of the world according to Eurofever data.

**Additional file 2: Table S2** Rare autoinflammatory diseases presented in Eurofever and JIR-cohort.

## Data Availability

The datasets used and/or analyzed during the current study are available from the corresponding author upon reasonable request.

## Abbreviations

PRES: Pediatric Rheumatology European Society
ERN RITA: European Reference Network on Rare Primary Immunodeficiency, Autoinflammatory, and Autoimmune Diseases
FMF: Familial Mediterranean fever
MKD: Mevalonate kinase deficiency
PFAPA: Periodic fever, aphthous stomatitis, pharyngitis, and adenitis syndrome
SAID: Systemic autoinflammatory disease
TRAPS: Tumor necrosis factor receptor-associated periodic syndrome
CAPS: Cryopyrin-associated periodic syndrome ¼ NLRP3-associated autoinflammatory disease (NLRP3-AID)
SoJIA: Systemic onset juvenile idiopathic arthritis
SURF: Syndrome of undifferentiated recurrent fever
CRMO: Chronic recurrent multifocal osteomyelitis
CNO: Chronic nonbacterial osteomyelitis
DADA2: ADA2 deficiency
DIRA: Deficiency of the IL-1 receptor antagonist
DITRA: Deficiency of the IL-36 receptor antagonist
HA20: A20 haploinsufficiency
PAPA: Pyogenic arthritis, pyoderma gangrenosum, and acne
PASH: Pyoderma gangrenosum, acne, and suppurative hydradenitis
MeRITA: Metadata registry for the ERN RITA
EAHC: Executive Agency for Health and Consumers
EDC: Electronic data capturing
GKJR: German Society for Pediatric Rheumatology
SOFREMIP: French-speaking pediatric rheumatology society
PRS: Pediatric Rheumatology Switzerland
