# Supplementary material for: Breaking down the fences among registries on autoinflammatory diseases: the E-Merge project
Source: Orphanet J Rare Dis. 2023 Jul 17;18:191. doi: 10.1186/s13023-023-02812-4 (PMC10353236; doi:10.1186/s13023-023-02812-4)
Supplement: Supplementary file 1 — Additional file 1: Table S1 Variables, suggested to be analysed from the registries (according to final meeting decision). Figure S1 Distribution of patients with autoinflammatory diseases within regions of the world according to Eurofever data. [file 13023_2023_2812_MOESM1_ESM.docx]

Suppl. table 1. Variables, suggested to be analysed from the registries (according to final meeting decision)

| Epidemiological data |
| --- |
| - Diagnose - Date of the diagnose, date of the disease onset, diagnose delay - Patient`s age and gender - Ethnicity and origin |
| Clinical data |
| - Core of the clinical signs available |
| Laboratory testing and treatment |
| - Genetic testing availability and panels used - Treatment (biologics) availability |

Suppl. figure 1. Distribution of patients with autoinflammatory diseases within regions of the world according to Eurofever data.
