# Supplementary material for: Breaking down the fences among registries on autoinflammatory diseases: the E-Merge project
Source: Orphanet J Rare Dis. 2023 Jul 17;18:191. doi: 10.1186/s13023-023-02812-4 (PMC10353236; doi:10.1186/s13023-023-02812-4)
Supplement: Supplementary file 2 — Additional file 2: Table S2 Rare autoinflammatory diseases presented in Eurofever and JIR-cohort. [file 13023_2023_2812_MOESM2_ESM.pdf]

Suppl. table 2. Rare autoinflammatory diseases presented in Eurofever and JIR-cohort

Eurofever (n = 4552)	JIR-cohort (n = 1897)
Blau syndrome (59)<br>PAPA (48)<br>DADA2 (46)<br>DIRA (3)<br>DITRA (1)<br>Majeed Syndome (3)<br>HA-20 (8)<br>CANDLE/PRAAS 1<br>NLRP12-related disease (18)<br>Schnitzler syndrome (14)<br>SAVI - STING-associated vasculopathy with onset in infancy (6)<br>Recurrent idiopathic pericarditis (6)<br>CAMPS (4)<br>COPA (1)<br>PLAID (1)	Blau syndrome (4)<br>PAPA (4)<br>DADA2 (12)<br>DIRA (5)<br>DITRA (1)<br>Majeed syndrome (3)<br>HA-20 (11)<br>CANDLE (2)<br>PASH (1)
